# Intraspinal Intradural Arachnoid Web at the Thoracic Level: A Case Report

**DOI:** 10.7759/cureus.67817

**Published:** 2024-08-26

**Authors:** Wahab Moustafa, Jörg Silbermann, Salah Maskoud, Mohamad Kinan Sultan, Amr Badary

**Affiliations:** 1 Department of Neurotraumatology and Spine, Stiftung Rehabilitation Heidelberg (SRH) Wald-Klinikum Gera, Academic Hospital of Jena University, Gera, DEU; 2 Department of Neurosurgery, Darmstadt Clinical Center, Darmstadt, DEU

**Keywords:** thoracic spine, myelopathy, intradural mass, hemilaminectomy, arachnoid web

## Abstract

Arachnoid web is a rare condition, which can cause significant neurological symptoms due to spinal cord compression. This case highlights the clinical presentation, surgical management, and pathology of an arachnoid web, contributing to the understanding of this rare condition. A 68-year-old female presented with diffuse pain syndrome, gait disturbance, and left leg pain. Clinical-neurological examination indicated myelopathy, confirmed by magnetic resonance imaging (MRI) revealing an intraspinal intradural mass at T4 with early signs of myelopathy. Surgical removal of the mass was performed via right-sided hemilaminectomy and microsurgical excision. Intraoperative findings included a cerebrospinal fluid (CSF)-filled pouch beneath an abnormal arachnoid membrane. Pathological analysis identified connective tissue fragments consistent with an arachnoid cyst, without atypia or inflammation. Postoperative recovery showed reduced leg pain and improved mobility.

## Introduction

The arachnoid web, a rare and poorly understood spinal cord condition, has been documented in only 41 cases in the literature as of the last systematic review in 2019 [1]. It involves the formation of an abnormal thickening or web-like structure of the arachnoid membrane. This thickening can exert pressure on the spinal cord, leading to a range of neurological symptoms [2]. Because of its rarity and the subtlety of its symptoms, the arachnoid web is often misdiagnosed or overlooked in clinical practice [3].

Patients with an arachnoid web typically present with symptoms related to spinal cord compression. These symptoms can include diffuse pain, difficulty walking, and changes in sensation or motor function [4]. In many cases, patients may experience a gradual onset of symptoms, which can progress over time if the condition is not treated [5]. Early diagnosis is crucial to prevent permanent neurological damage and improve the patient's quality of life.

Imaging studies, particularly magnetic resonance imaging (MRI), play a crucial role in diagnosing arachnoid webs. MRI can provide detailed images of the spinal cord and reveal the presence of a web-like structure. A characteristic feature of the arachnoid web on MRI is the "scalpel sign," which appears as a dorsal indentation of the spinal cord. This sign helps differentiate arachnoid webs from other intradural pathologies such as tumors or cysts [6].

The primary treatment for an arachnoid web is surgical intervention. The goal of surgery is to decompress the spinal cord by removing the thickened arachnoid membrane. This procedure typically involves a hemilaminectomy, which is the removal of a portion of the vertebral bone to access the spinal cord [7]. Once the abnormal membrane is exposed, it is carefully dissected and removed to relieve the pressure on the spinal cord. Successful surgical intervention can lead to significant improvement in symptoms and prevent further neurological deterioration.

Histological examination of the excised tissue is essential to confirm the diagnosis of an arachnoid web. Pathological findings usually include membranous fragments of connective tissue with very low cellularity, devoid of atypia and inflammation [8].

By sharing this case, we aim to contribute to the limited literature on this rare condition and provide insights into its diagnosis and treatment. Understanding the clinical and radiological characteristics of arachnoid webs can help clinicians make timely diagnoses and offer appropriate treatment to improve patient outcomes.

## Case presentation

Clinically, a 68-year-old female presented with diffuse pain, primarily in her left leg, and gait disturbances. The pain was diffuse rather than radiculopathic. Neurological examination showed heightened reflexes in the lower extremities and muscle weakness. The Romberg test revealed a significant imbalance, indicating posterior spinal cord involvement. Muscle strength, assessed using the Medical Research Council (MRC) Manual Muscle Testing scale, was found to be between grades 3 and 4 in the hip flexors, knee extensors, and ankle dorsiflexors.

**Figure 1:**
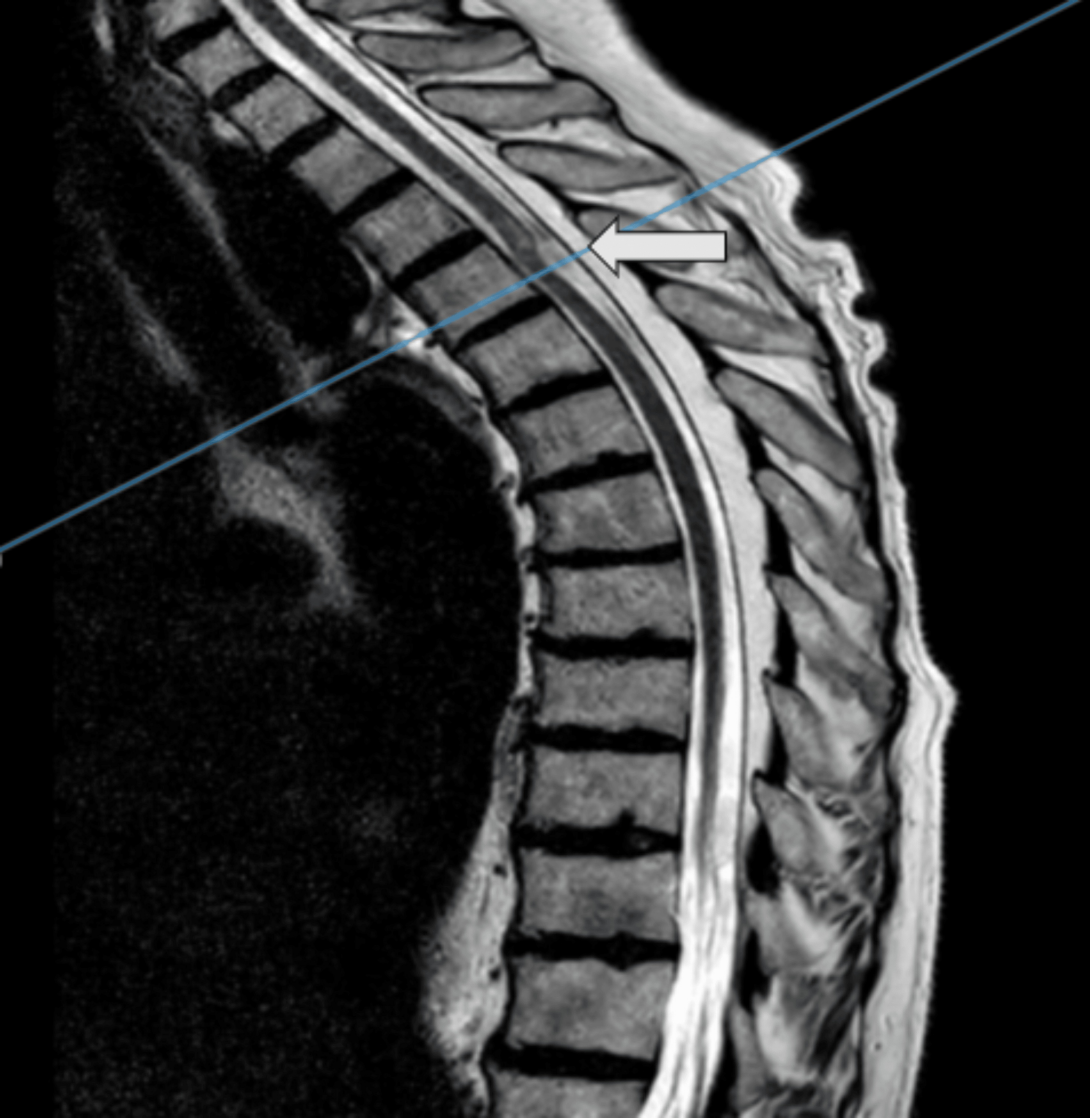
Preoperative T2-weighted MR image in sagittal view (arrow showing the intraspinal mass at the level of T4) MR: magnetic resonance

**Figure 2:**
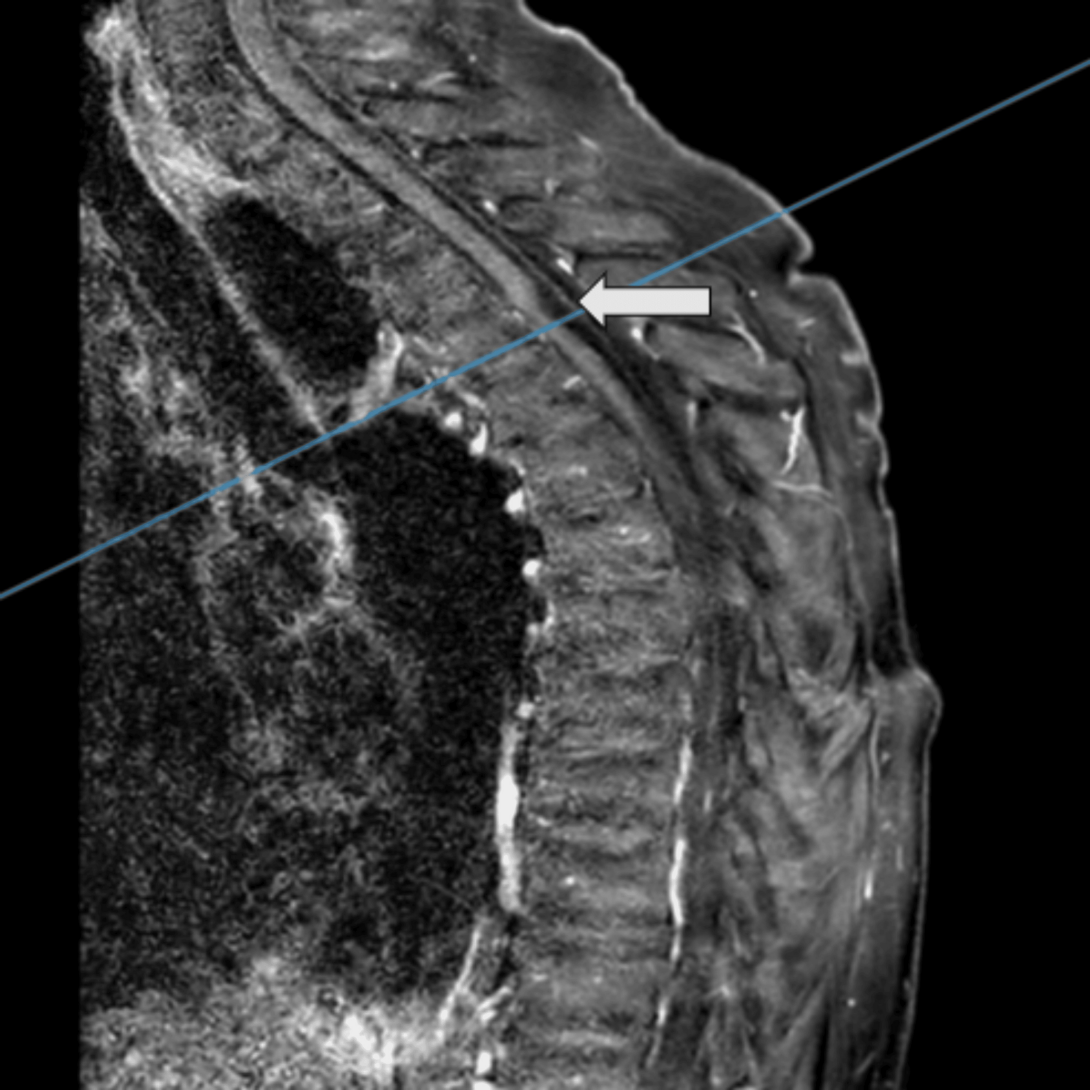
Preoperative T1-weighted MR image with contrast in sagittal view (arrow showing the intraspinal mass, compressing the spinal cord at the T4 level) MR: magnetic resonance

Radiologically, an MRI of the thoracic spine, utilizing T1- and T2-weighted sequences, revealed an intraspinal, intradural arachnoid web at the level of the fourth thoracic vertebra (T4), with early signs of myelopathy above the affected area (Figure 1 and Figure 2).

Surgical intervention was performed via a right-sided hemilaminectomy at the T4 vertebral level, followed by microsurgical excision of the intraspinal, intradural mass, which was then submitted for histological analysis.

During the procedure, we identified an intradural, pulsatile, bulging pouch filled with cerebrospinal fluid (CSF) beneath an abnormally thickened arachnoid membrane. This thickening was constricting the dorsal surface of the spinal cord. The arachnoid wall (AW) was carefully dissected from the pia mater at multiple points until normal, unaffected tissue was reached, restoring normal CSF circulation and relieving the compression.

Postoperative course

During the hospital stay, the patient experienced a reduction in leg pain and improved mobility.

Pathology report

The specimen reveals membranous fragments of connective tissue with very low cellularity, devoid of atypia and inflammation. Part of the specimen has a covering of accentuated, atypia-free nucleated cells. These findings are generally consistent with a component of an arachnoid cyst (clinical suspected diagnosis). However, the overall findings are non-specific. No malignancy or significant inflammatory infiltration was observed.

## Discussion

Arachnoid webs are rare spinal pathologies that can cause significant neurological deficits due to their compressive effect on the spinal cord. The clinical presentation of an arachnoid web often includes pain, gait disturbances, and sensory or motor deficits [9], as seen in our patient. Myelopathy was evident through heightened reflexes and MRI findings, underscoring the importance of imaging in the diagnosis of spinal cord compression disorders. These symptoms align with findings from a systematic review, which reported that weakness was present in 67% of cases and lower extremity involvement in 81% [10]. The positive Romberg test suggests posterior spinal cord involvement, consistent with the review's observation of thoracic spine localization for arachnoid webs [10].

MRI is the gold standard for diagnosing arachnoid webs, as it provides detailed images of the spinal cord and surrounding structures. The typical radiological sign, known as the "scalpel sign," was present in our case, characterized by a dorsal indentation of the spinal cord at the T4 level. This finding is crucial for distinguishing arachnoid webs from other intradural pathologies such as tumors or cysts​​ [11]. The review confirms that arachnoid web of the spine (AWS) is exclusively located in the thoracic spine and is usually positioned dorsally relative to the spinal cord [12]. Although our patient did not have a syrinx, which was found in 67% of cases in the review, she did exhibit early myelopathic changes [13].

Surgical management of arachnoid webs involves decompression of the spinal cord by removing the abnormal arachnoid membrane [14,15]. In our case, a right-sided hemilaminectomy at T4 allowed for adequate exposure and excision of the arachnoid web. The intraoperative findings of a pulsatile CSF-filled pouch and thickened arachnoid membrane were consistent with previous reports​​ [16,17]. The meticulous detachment of the arachnoid wall from the pia mater was crucial in restoring normal CSF passage in the subarachnoid space and alleviating spinal cord compression.

The surgical approach, involving a laminectomy with intradural excision, aligns with the technique used in 86% of cases as documented in the review [18]. Postoperatively, the patient experienced reduced leg pain and improved mobility, consistent with the review's finding that 91% of patients reported neurological improvement following surgery [18]. These outcomes underscore the effectiveness of the surgical technique and its consistency with established results.

The postoperative course of our patient showed significant improvement, with reduced leg pain and improved mobility. This outcome highlights the effectiveness of surgical intervention in relieving symptoms and improving the quality of life in patients with arachnoid webs. Previous studies have also reported favorable outcomes following surgical decompression of arachnoid webs, further supporting the benefits of timely surgical management​​ [19].

Pathological examination of the excised tissue is essential for confirming the diagnosis and excluding other conditions. In our case, the specimen revealed membranous fragments of connective tissue with very low cellularity, devoid of atypia and inflammation, consistent with an arachnoid cyst. These findings are in line with previous histopathological reports of arachnoid webs, which describe similar tissue characteristics​​ [8]. The absence of malignancy or significant inflammatory infiltration further supports the benign nature of arachnoid webs​​.

Future research should focus on long-term outcomes and potential complications following surgical treatment of arachnoid webs. Further studies are needed to explore the underlying pathophysiology and genetic factors contributing to the development of arachnoid webs. A comprehensive understanding of these aspects will aid in developing targeted therapies and improving patient care.

## Conclusions

This case study describes the diagnosis, surgical treatment, and outcomes of a 68-year-old female with an arachnoid web at T4. Her symptoms were effectively managed with surgery, guided by an MRI showing the "scalpel sign." The procedure led to significant improvement, and pathology confirmed an arachnoid cyst. This case underscores the importance of early diagnosis and surgery in managing arachnoid webs and adds valuable insight to the limited literature on this rare condition.
